# Effects of the degree of freedom and assistance characteristics of powered ankle-foot orthoses on gait stability

**DOI:** 10.1371/journal.pone.0242000

**Published:** 2020-11-10

**Authors:** Ho Seon Choi, Yoon Su Baek

**Affiliations:** School of Mechanical Engineering, Yonsei University, Seoul, Republic of Korea; University rehabilitation institute, SLOVENIA

## Abstract

We studied the use of powered ankle-foot orthoses (PAFOs) and walking stability of the wearers, focusing on the ankle joint, which is known to play a critical role in gait stability. Recognizing that the subtalar joint is an important modulator of walking stability, we conducted the walking experiment on a treadmill by applying varying assistance techniques to the 2-degree-of-freedom (DOF) PAFO, which has the subtalar joint as the rotating axis, and the commonly used 1-DOF PAFO. The participants were 8 healthy men (mean±SD: height, 174.8±7.1 cm; weight, 69.8±6.5 kg; and age, 29.1±4.8 years) with no history of gait abnormality. Center of pressure (COP) was measured with an in-shoe pressure sensor, and stability was estimated on the basis of the angular acceleration measured with the inertial measurement unit attached to the trunk. The experimental results of the 2-DOF PAFO, with or without assistance, showed a significantly higher stability than those of the 1-DOF PAFO (up to 23.78%, p<0.0326). With the 1-DOF PAFO, the stability deteriorated with the increase in the degree of assistance provided. With the 2-DOF PAFO, this tendency was not observed. Thus, the importance of the subtalar joint was proven using PAFOs. The mean position analysis of the COP during the stance phase confirmed that the COP highly correlated with stability (Pearson correlation coefficient: −0.6607). Thus, we conclude that only the 2-DOF PAFO can maintain walking stability, regardless of the assistance characteristics, by preserving the COP in the medial position through eversion. Awareness regarding the role of the subtalar joint is necessary during the manufacture or use of PAFOs, as lack of awareness could lead to the degradation of the wearer’s gait stability, regardless of effective assistance, and deteriorate the fundamental functionality of PAFO.

## Introduction

Exoskeletons, also called wearable suits, are a type of robot designed to be worn by a person to provide functions that assist in human movement [1]. These robots are being actively developed in the field of rehabilitation engineering and are usually used for elderly people or patients who have lost their gait ability. They can be used as rehabilitation exercise devices to improve or relearn walking skills and have been developed and utilized for various other purposes [2–7]. Among these devices, powered ankle-foot orthoses (PAFOs) are used to assist ankle joints [8]. The ankle joint has an important role in walking assistance because it is the rotation axis connecting the calf and foot, which not only exerts the largest torque when walking but also controls the movement of the foot, and acts as an end-effector by directly contacting the ground [9]. The ankle joints consist of talocrural and subtalar joints, which are responsible for propulsion through dorsi/plantar flexion and adaptation to the ground through inversion/eversion, respectively [10].

The users of PAFOs are people who have weakened muscles because of aging or disease. Usually, the fall risk of these individuals is increased due to weakened muscle strength [11], so they supplement their loss of muscle power with assistance of PAFOs to be free from the danger of falls and conduct normal walking. Previous research has focused on the talocrural joint, which is the rotation axis of dorsiflexion that prevents foot drop during the swing phase or of plantar flexion, which is responsible for the push-off during the gait cycle [12–14]. However, the lack of research on the subtalar joint, which keeps the body balanced and stable when walking, can be contradictory to the development of PAFOs.

Stability is defined as resistance to biomechanical changes [15]. It means that the center of mass (COM), which is regarded as the point of action for the resultant perturbation, should be kept in a stable position or trajectory for balance. Balance is defined as the ability to keep the COM in the base of support. Thus, stability can be an index for balance and should be secured for conducting balanced motion [16]. For that reason, postural sway, which is the movement of the COM, which is the trunk in this case [17], is used to measure clinical changes of balance [18–20]. Thus, for maintaining balanced walking, stability should be maintained during the gait cycle, and the subtalar joint is the key element of PAFOs to help wearers maintain the aforementioned functions.

The subtalar joints maintain the postural control of the body in the frontal plane by adjusting the position of the center of pressure (COP) through rotation of the foot [21–23]. The location of the COP is an important indicator of stability during gait, that is, gait stability, and influences the calculation of the extrapolated COM and margin of stability [24, 25]. Therefore, in the absence of the subtalar joints, the position and speed of the COP are limited, resulting in increased postural sway of the frontal plane during the one-leg stance. Hoogveliet et al. demonstrated this through experiments using ankle braces [23]. Thus, if PAFOs do not have a subtalar joint as the rotational axis, it might generate postural problems in wearers.

Another problem results from the absence of a subtalar joint. Bok et al. found that factors related to the deterioration of stability due to aging include the weakening strengths of the muscles responsible for eversion by the subtalar joint and the consequent reduction in range of motion [26]. In other words, the strength of the peroneus longus muscles is weakened due to aging, which prevents these muscles to play their role as an evertor sufficiently during the gait cycle. In fact, the three-dimensional moment of eversion was significantly reduced due to aging in elderly individuals as compared with healthy young individuals [27]. As a result, in elderly people, the walking skills are modified by factors such as increased step width and decreased walking speed to avoid falls, but the reduced mediolateral stability is not avoided. Various factors contribute to this situation, of which the foot tilt strategy (FTS) is the major factor related to the subtalar joint. In the FTS, the evertor generates the stabilizing moment with rotation by the subtalar joint to compensate for the tilting moment resulting from the offset between the foot plantar COP and the projection of the COM [22]. Thus, humans can keep their body in a stable state. When this process cannot be conducted normally, stability would deteriorate, and a fall could occur.

Falls occur when gait stability is lost during walking [11, 16]. This can be confirmed by comparing the gait stability of fall-prone elderly people, healthy elderly people, and young individuals. Liu et al. assessed fall risk by using the inertial measurement unit (IMU) in the three populations [19]. The fall-prone elderly people had significantly greater COP speed, mediolateral and anteroposterior accelerations of the trunk, and maximum Lyapunov exponent. In addition, Howcroft et al. also proved the difference in stability between fall-prone and healthy elderly people during dual-task walking by using a triaxial accelerometer and pressure-sensing insole [20].

These situations show the reason why the subtalar joints should not be excluded from the development of exoskeletons. However, the existing PAFOs have 1 degree of freedom (DOF) with a talocrural joint. This means that they only have a fixed point of action and a constant force direction regardless of the type of actuator, which cannot help wearers keep their balance from external perturbations. Nevertheless, they are still useful because their focus was on the effective assistance for reducing metabolic rate to the maximum. However, if subjects with low stability due to the absence of a subtalar joint are given a certain direction of assistance limited to that subtalar joint, as discussed by Anam and Al-Jumaily, this can be perceived as a perturbation by the robot-human interface [28]. The muscle activity of the ankle might be reduced, and the overall stability is deteriorated, which affects other joints and elements. Therefore, these assistive techniques is unlikely easy to use owing to these shortcomings, as safety is the most important factor considering the characteristics of the subjects.

Therefore, we hypothesized that the presence of a subtalar joint in the PAFO design would improve the wearer’s stability. As the DOF increases, the wearer can achieve the FTS with assistance; through this, direction can be controlled. Thus, we expected that effective force transmission in the optimal direction with high stability is possible. Currently, 2-DOF PAFOs with actuatable talocrural and subtalar joints with pneumatic artificial muscles (PAMs) are available. In this study, by using the existing 2-DOF PAFOs and the additionally manufactured 1-DOF PAFOs, the effect of the subtalar joint in the PAFO and the change in the wearer’s gait stability resulting from various assistance techniques were analyzed to prove our hypothesis using the COP and stabilogram data.

## Materials and methods

This study compared the wearer’s stability, measured in unpowered and powered conditions, with the changes in the size and direction of assistance during walking with the 1- and 2-DOF PAFOs. The 2-DOF PAFO used for the experiments was developed by Choi et al. [29], and an additional 1-DOF PAFO lacking a subtalar joint was developed for this study.

### The 2-DOF powered ankle-foot orthosis

**Fig 1:**
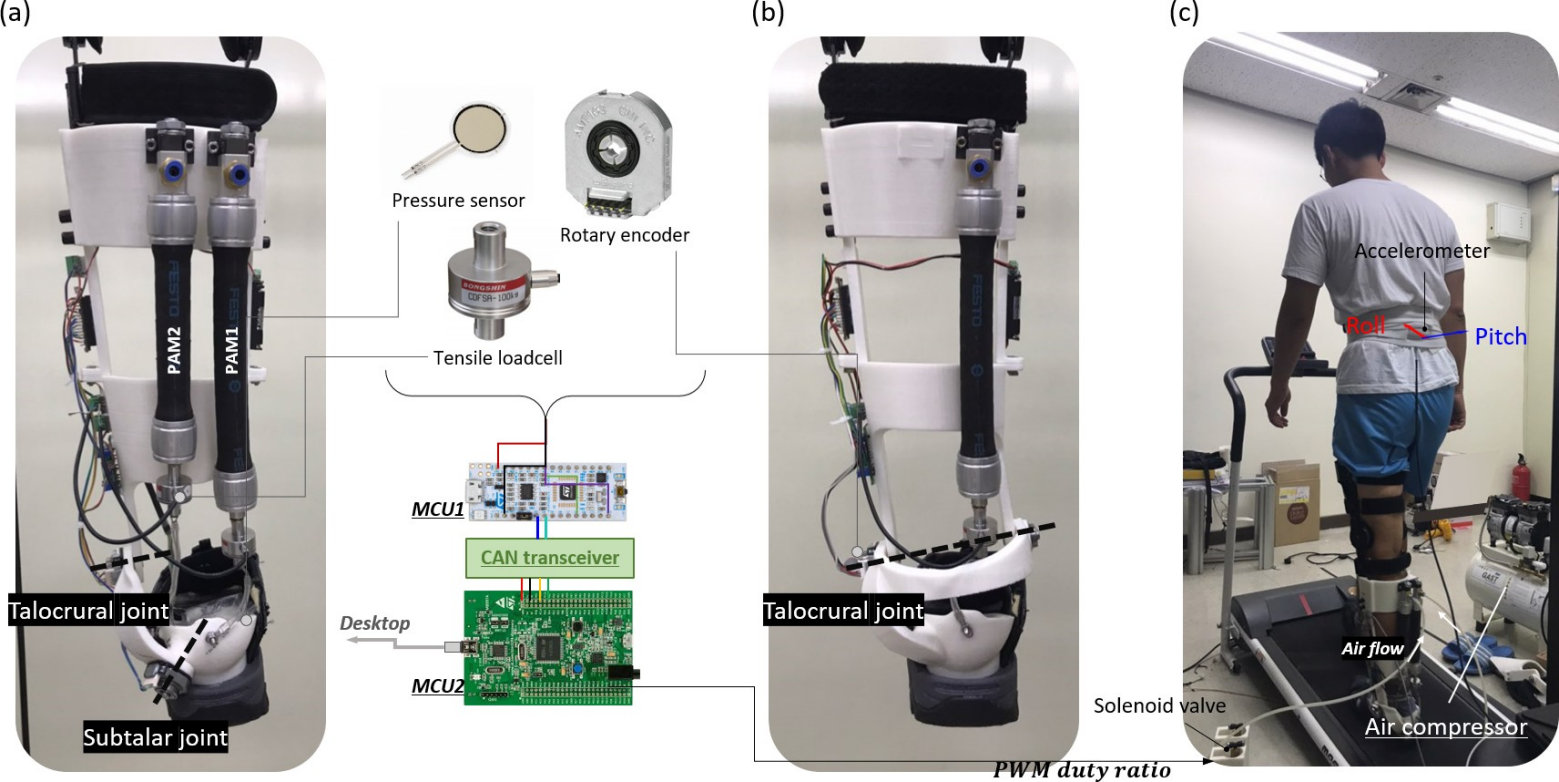
The overall system and experimental environment. (A) and (B) represent the 2- and 1-DOF PAFOs, respectively. (C) shows the experimental setup. The subject wore an in-show pressure sensor and IMU on the trunk and walked on the treadmill. The figure also shows the flow of data. The signals detected by the PAFO sensors are measured by micro controller unit 1 (MCU1; STM Nucleo board) mounted on it and transferred to MCU2 (STM Discovery board) via CAN communication. MCU2 generates a PWM signal for solenoid valve control and enables serial debugging in the desktop through simultaneous serial communication. And the individual in this figure has given written informed consent (as outlined in PLOS consent form) to publish these case details.

**Fig 2:**
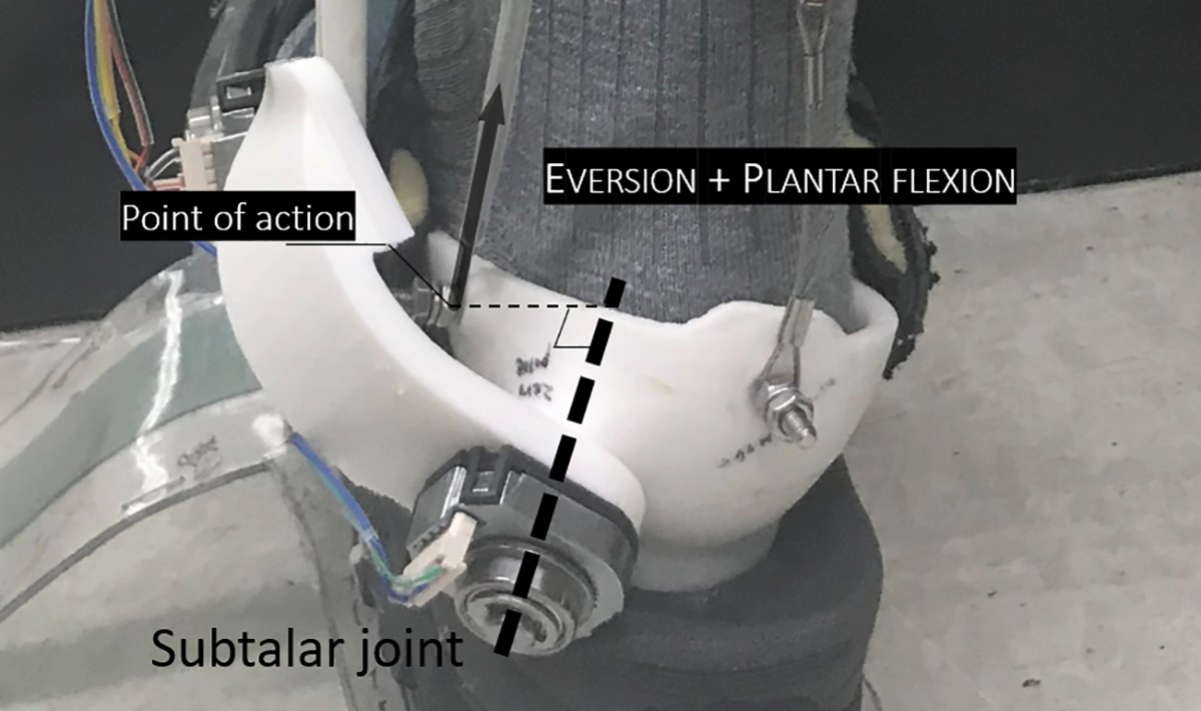
Contraction of PAM2 for simultaneous eversion and plantar flexion by rotation of the subtalar and talocrural joints.

The 2-DOF PAFO used in this study divides the ankle joint into 2 axes of rotation, as shown in Fig 1A and explained by Choi et al [29]. Spatial formulas for the talocrural and subtalar joints were calculated using the mean of the individual values based on the anthropometric data of Isman and Inman [10]. The robot is interfaced with humans at the feet, calves, and thighs as shown in Fig 1C. Force sensitive resistors (FSRs) are located under the foot to determine the heel strike, and each joint is equipped with an encoder to measure the angle of rotation. Two PAMs are used as the actuator, one acting as the soleus to aid plantar flexion and the other acting as the peroneus longus to assist eversion with plantar flexion as shown in Fig 2. Both PAMs are connected to the solenoid valves, which are controlled by pulse width modulation (PWM) signals and supplied with air pressure, maintained at 6 bar, from the air compressor and regulator. The PAMs and frame are connected by wires, and in the middle of the wires, tensile loadcells are attached to measure the force transmitted. All sensing values are measured on the STMicroelectronics (STM) Nucleo board and transmitted to the STM Discovery board via controller area network (CAN) communication. The discovery board determines the stage of the gait cycle based on the acquired values and sends the certain duty ratio calculated for the contraction of the PAMs to the solenoid valve to implement PAFO assistance. A more detailed explanation is given in the paper by Choi et al. The 1-DOF PAFO was manufactured by simply removing the subtalar joint, as shown in Fig 1B.

### Assistance techniques

Various assistance techniques were developed and applied to compare the stability between wearing the 1- and 2-DOF PAFOs. The types of assistance techniques are listed in Table 1. For the reference data, the experiments were conducted in the unpowered state without assistance.

**Table 1:** Types of assistance technique for PAFOs.

DOF	Duty ratio [*%*]		Abbr.	PAM1			PAM2		
	PAM1	PAM2		*f*_*peak*_[*N*]	*t*_*ass*_[*%*]	*t*_*ppo*_[*%*]	*f*_*peak*_[*N*]	*t*_*ass*_[*%*]	*t*_*ppo*_[*%*]
1	0	0	1 DOF UNP	-	-	-	-	-	-
	30	0	1 DOF A	59.73 (±25.47)	21.3 (±6.8)	47.8 (±3.8)	-	-	-
	60	0	1 DOF B	93.51 (±20.92)	15.9 (±4.6)	46.0 (±6.3)	-	-	-
	90	0	1 DOF C	132.42 (±22.29)	12.1 (±3.4)	43.6 (±11.0)	-	-	-
2	0	0	2 DOF UNP	-	-	-	-	-	-
	30	0	2 DOF A1	60.03 (±19.40)	21.5 (±9.1)	48.5 (±4.3)	-	-	-
	30	30	2 DOF A2	20.76 (±13.40)	18.9 (±6.9)	48.5 (±3.4)	79.06 (±26.06)	27.8 (±6.6)	49.6 (±3.8)
	60	0	2 DOF B1	110.08 (±33.43)	17.1 (±3.8)	51.4 (±4.4)	-	-	-
	60	60	2 DOF B2	57.45 (±38.04)	17.5 (±5.0)	48.9 (±4.4)	76.21 (±24.45)	23.1 (±4.4)	49.9 (±5.4)
	90	0	2 DOF C1	141.97 (±64.99)	14.0 (±5.5)	50.3 (±4.2)	-	-	-
	90	90	2 DOF C2	114.83 (±57.11)	14.5 (±6.9)	47.6 (±7.6)	60.83 (±18.87)	26.9 (±9.5)	47.1 (±6.0)
	90	90	2 DOF C3	82.93 (±61.25)	17.8 (±8.2)	48.1 (±5.4)	107.30 (±22.41)	16.6 (±4.2)	49.6 (±3.9)

*f*_*peak*_, *t*_*ass*_, *t*_*ppo*_ mean magnitude of peak force, assistance timing and positive power onset, respectively. And the values in parentheses indicate standard deviations.

#### The 1-DOF powered ankle-foot orthosis

**Fig 3:**
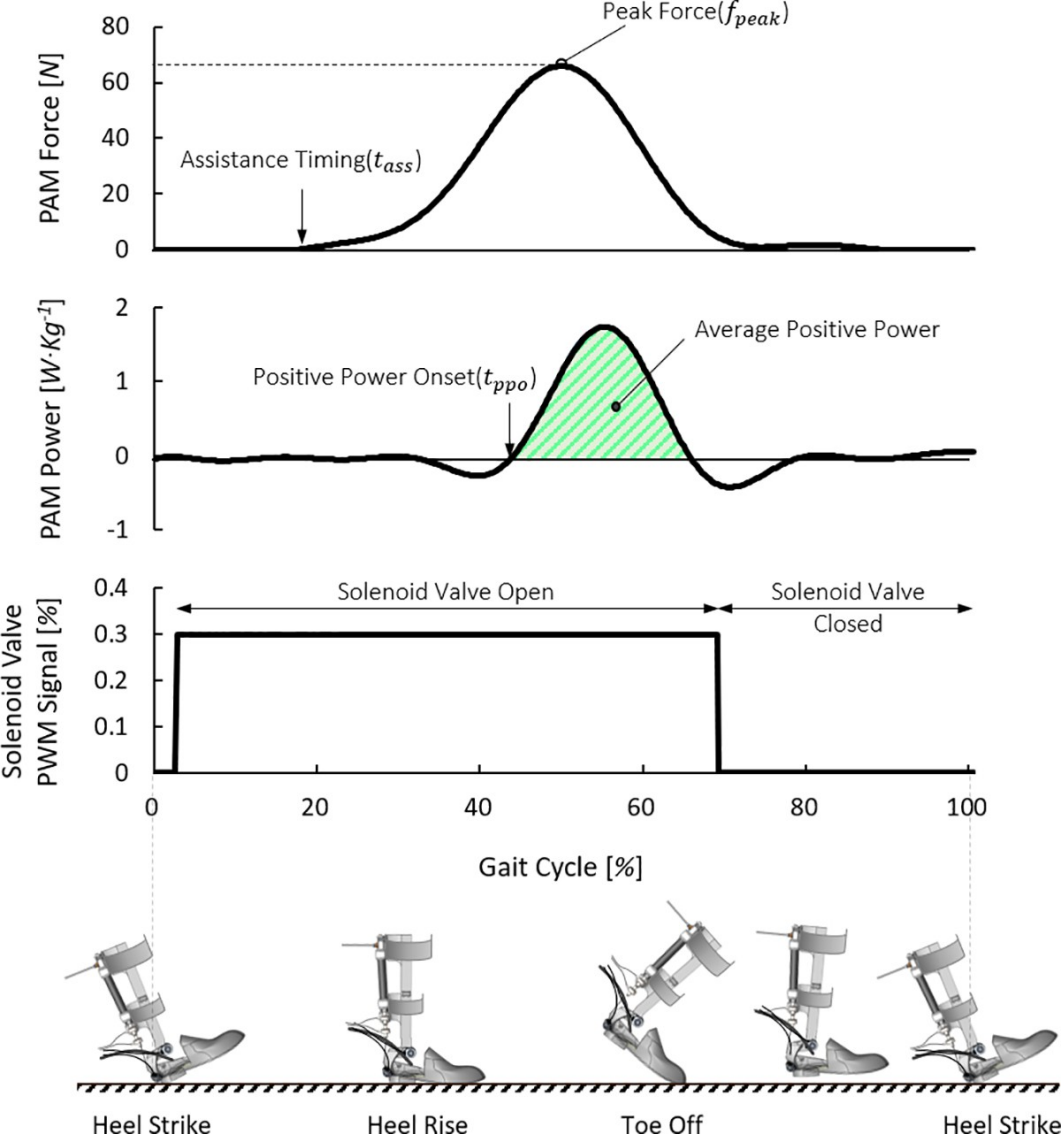
Characteristics of the assistance techniques. This figure describes the characteristics of P-bcs such as assistance timing, peak force, and onset of positive power. The illustrations of the foot posture on the ground during gait cycle helps the understanding of the process of assistance.

We used a commonly used phase-based controller (P-bc), known as an ankle positive power inspired technique for a 1-DOF PAFO [8]. The P-bc is an assistance technique commonly used with PAFOs that have a PAM as an actuator to maximize the duty ratio of the solenoid valve at a certain timing to provide intensive assistance in the period when the ankle power is positive during the gait cycle [30–33]. The period with positive values occurs when the ankle joint performs plantar flexion. We used the FSR sensor to determine the time point. Among experiments of PAFOs with PAMs, the onset timing of positive power was between 13% and 54% of the gait cycle [33], but high effectiveness of assistance was most frequently achieved between 40% and 50% [32, 34, 35]. We used the stage of the gait cycle, detected by the FSR sensor, to provide 3 duty ratios (30%, 60%, and 90%) at the heel strike and then stopped the pulse at the toe-off time. This results in an onset timing of positive power between 40% and 50% for all the cases are shown in Table 1, which also contains the magnitudes of peak force and assistance timing for each duty ratio, which is explained in detail in Fig 3.

#### The 2-DOF powered ankle-foot orthosis

The same P-bc was used for comparison with the 1-DOF PAFO. However, in addition to PAM1, the 2-DOF PAFO has PAM2, which acts as the peroneus longus, allowing for a wider variety of controls. As shown in Table 1, the control methods of the P-bcs for the 2 PAMs are classified. The duty ratio of PAM2 does not exceed the size of PAM1, and in both cases, 3 duty ratios were used, and a total of 8 P-bcs were applied.

### Experimental protocol

The number of subjects was determined by referring to the number of previously conducted PAFO experiments [33–36]. A total of 8 healthy men (mean ± SD: height, 174.8 ± 7.1 cm; weight, 69.8 ± 6.5 kg; and age, 29.1 ± 4.8 years) with no history of gait abnormality participated in the experiment, which was approved by the institutional review board of Yonsei University (7001988-202003-HR-833-03). Proper written consents were obtained from the participants before the experiment. They were recruited on the campus of Yonsei University regardless of gender. The participants wore a pressure-sensing insole to measure the COP and an IMU to measure the postural sway of the trunk, in addition to the PAFO, as shown in Fig 1C. The subjects walked on the treadmill at a speed of 1.2 m/s, and various assistance techniques were applied. The assistance techniques were applied to the left foot only, and the right PAFO was used without actuation. As with other exoskeleton experiments, we gave the wearer some time to get used to the robot to allow for reliable experimental data measurements and gave the wearers enough rest time between experiments to avoid fatigue. Specifically, before the experiments, they performed treadmill walking two times within 30 minutes to get used to each robot with assistance [35, 37], and 3 minutes was provided between each set of 50 steps. When the experiments with 1-DOF PAFO was finished and before entering the second experiment with 2-DOF PAFO, they took a 10-minute rest to prevent fatigue. The total walking time was around 2 hours, and all the assistance techniques were evaluated thrice over 50 steps each.

### Measurements

#### Center of pressure

**Fig 4:**
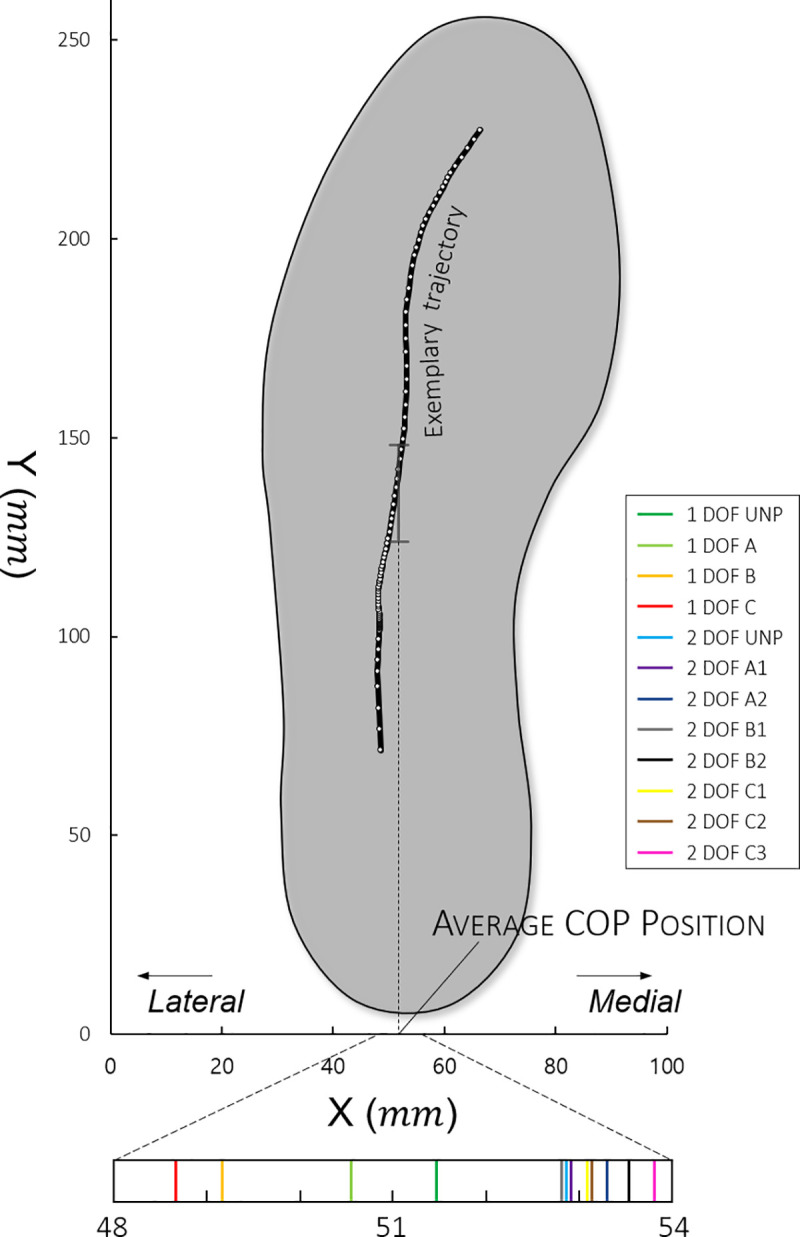
Methods for calculating the average COP position and examples with 1- and 2- DOF PAFOs. The trajectory was interpolated to increase the data to 100 points during the stance phase.

We measured the COP using a pressure-sensing insole (MP2512PLUS; Kitronyx Inc, Seoul, Korea) that can be worn inside the shoe and obtained the data at a frequency of 50 Hz. The data were normalized by the gait cycle using the FSR foot sensor, and the average was calculated and represented as a single trajectory. After this, we calculated the average COP position (*COP*_*Avg*_), which is the average of its medial-lateral displacement values as in Fig 4.

#### Eversion angle

After measuring the angle change during the gait cycle from the rotary encoder attached to the subtalar joint of the PAFO, the average (*θ*_*Avg*_) was calculated to determine the degree of quantitative eversion.

#### Positive power of the PAM

By substituting the rotation angle of the 2 joints into the Rodrigues’ rotation formula used by Choi et al., the change in the length of the PAM can be calculated. The Rodrigues’ rotation formula determines the location at which a point is rotated at an angle with respect to an arbitrary straight line in space [38]. The contraction rate of the PAM can be calculated from the change in length, and the power can be obtained by multiplying the magnitude of the force obtained from the load cell. Normally, the average value of positive power applied to the human body during the gait cycle is proportional to the decrease in electromyography (EMG) activity or metabolic cost [39]. The average positive power is the same as the work applied to the human body. Thus, to compare the difference in the work applied to the human body, we also calculated and showed the average value of the positive area (*P*_*Avg*_) in the power curve for each experiment.

#### Stabilograms

**Fig 5:**
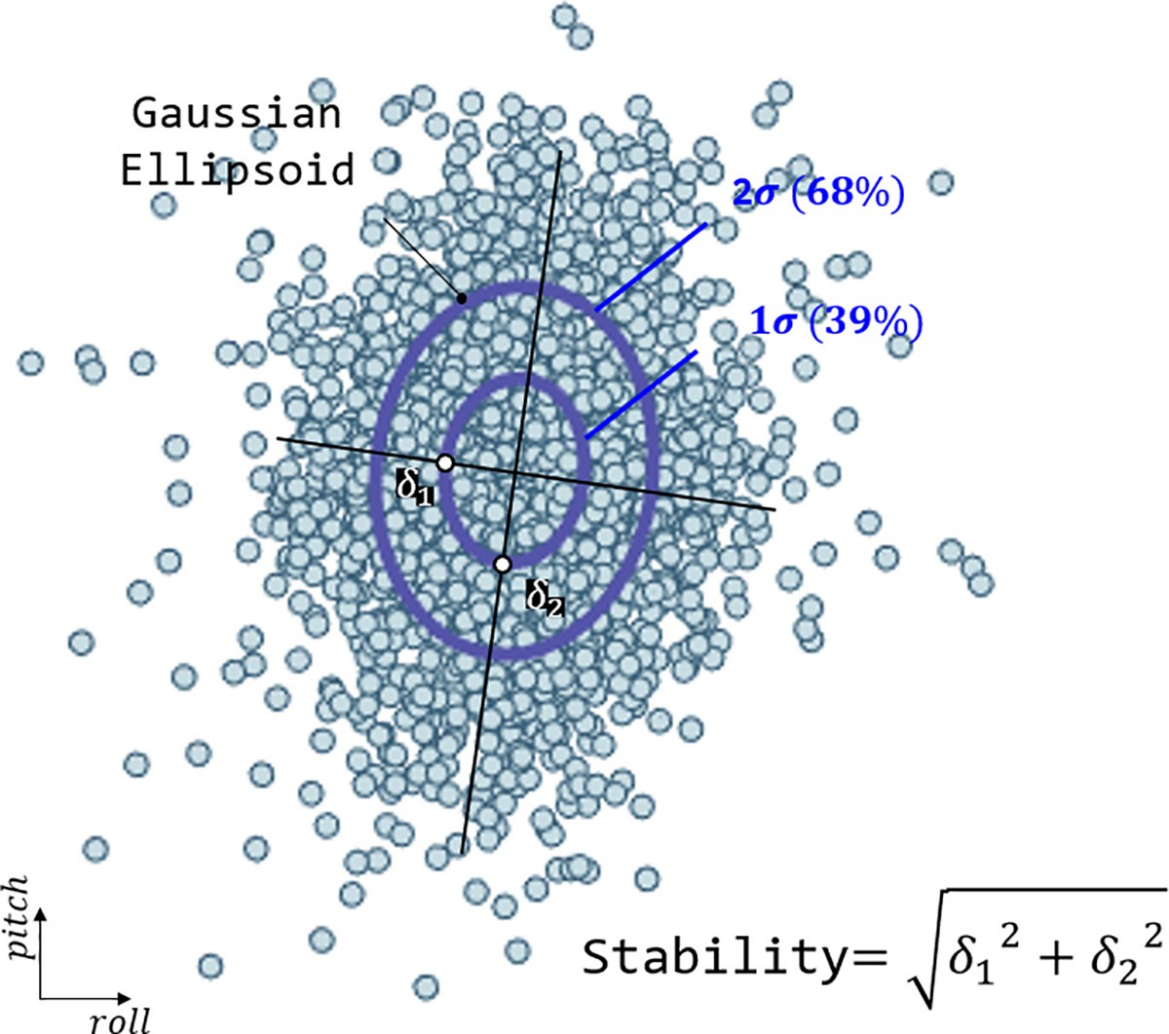
Stabilogram of the trunk angular acceleration using a density plot. Converting to a Gaussian ellipsoid through covariance calculation makes the comparison easier, as shown on the right. In the figure, *δ*_1_ and *δ*_2_ represent 2 standard deviations of covariance and are used as vertices of the Gaussian ellipsoid.

The stabilograms obtained in this study consisted of the angular acceleration values of the IMU attached to the trunk. According to Kang et al. [17], maintaining the balance of the upper body is important to avoid falling during any form of movement, including walking, and this balance is determined by the movement of the lower limb. Therefore, as in other studies, because the trunk has the smallest change in external perturbation and represents the COM of the whole body, the IMU was attached to it, and stability was judged on the basis of the detected movement [17, 19]. The values obtained from the IMU were converted into Euler angles using a low-pass filter and a complementary filter. We used the Newton difference quotient with backward differences to convert the angles of the roll (medial/lateral) and pitch (anterior/posterior) to angular accelerations. These were represented as a stabilogram on a two-dimensional (2-D) plot, as shown by Seimetz et al. So far, studies have used the maximum radius to compare these stabilograms [40]. However, this comparison is not reliable, and we need stochastic values. Therefore, we applied the Gaussian ellipsoid [41]. As shown in Fig 5, we can calculate the covariance of 2-D angular acceleration data and draw an ellipsoid with vertices of eigenvalue, which means 2 standard deviations. Usually, 1δ, 2δ, and 3δ are used to represent 3 Gaussian ellipsoids containing 39%, 68%, and 99% of the data, respectively. This enables a quantitative and reliable comparison of stabilograms. We compared the overall stability by calculating the root mean square values (*δ*_*RMS*_) with two 1δs representing the roll and pitch directions, respectively. So, if stability deteriorated, then the *δ*_*RMS*_ will be bigger than before.

### Statistical analyses

We compared the average COP position (*COP*_*Avg*_), average eversion angle (*θ*_*Avg*_), stabilogram (*δ*_*RMS*_), and average positive power (*P*_*Avg*_), and calculated the average and standard deviation for each experimental parameter in Table 2. In the case of *COP*_*Avg*_, the data were expanded by interpolating 100 data recorded during the stance phase to increase reliability. The rest of the data were also normalized to the 100 data for the entire gait cycle and compared. To compare the above-mentioned results according to the DOF and assistance techniques, the homogeneity and heteroscedasticity of variance were judged using the f-test between a total of 12 test results, and the difference between the data was verified using a *t* test for each case. The p value, the result of the *t* test, is shown for easy comparison between the experiments through the p-table. For comparison between the 12 cases, *δ*_*RMS*_ was converted to the rate with respect to the unpowered condition with the 2-DOF PAFO, and the average positive power was divided by the participant’s weight. The average eversion angle and COP position were used as they were and included in the S1 Dataset. The level of significance was determined on the basis of the false discovery rate (FDR) [42]. In addition, Pearson correlation coefficients (PCCs) were calculated between *P*_*Avg*_−*θ*_*Avg*_, *θ*_*Avg*_−*COP*_*Avg*_, *COP*_*Avg*_−*δ*_*RMS*_, and *P*_*Avg*_−*COP*_*Avg*_.

**Table 2:** Experimental results of all the participants.

			1 DOF				2 DOF								Unit
			UNP	A	B	C	UNP	A1	A2	B1	B2	C1	C2	C3	
***δ***_***RMS***_		Avg.	8.055	8.306	8.583	8.754	7.339	7.057	6.672	7.510	7.037	6.957	6.892	6.754	$\ddot{\theta }$
		Std.	1.051	1.372	1.177	1.234	1.166	1.049	0.752	1.089	0.908	0.765	0.779	0.879	
***COP***_***Avg***_		Avg.	51.433	50.688	49.172	48.731	52.913	52.827	53.267	52.882	53.533	53.151	53.066	53.623	*mm*
		Std.	1.571	1.843	2.643	2.756	1.928	2.078	1.717	1.719	1.309	1.766	2.017	1.810	
***θ***_***Avg***_		Avg.					0.160	0.356	1.339	-0.070	1.577	0.549	1.756	2.640	*θ*
		Std.					1.321	1.830	1.956	1.959	1.651	1.523	1.201	1.052	
***P***_***Avg***_	(1)	Avg.		0.027	0.041	0.055		0.022	0.009	0.026	0.012	0.029	0.020	0.014	*W/kg*
		Std.		0.015	0.015	0.022		0.007	0.005	0.010	0.008	0.015	0.015	0.014	
	(2)	Avg.							0.019		0.015		0.013	0.023	
		Std.							0.009		0.007		0.005	0.008	

The bigger the magnitude of *δ*_*RMS*_, the much worse the stability is. And the direction of *COP*_*Avg*_ is that when its magnitude increase, it goes toward medial direction. Likewise, for *θ*_*Avg*_, the bigger value means that eversion was enhanced.

## Results

### Center of pressure

**Fig 6:**
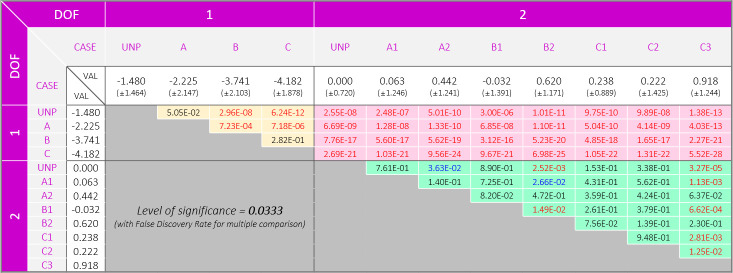
P-table of the center of pressure. The values with blue and red colors represent significant differences of p < 0.05 and p < 0.0333 for before and after correction, respectively. The significance level of 0.0333 was determined on the basis of FDR. The shadows colored in yellow, pink, and green represent comparisons between 1 DOF − 1 DOF, 1 DOF − 2 DOF, and 2 DOF − 2 DOF, respectively.

The results of the experiments with the 1-DOF PAFO show that the COP trajectories were generally more localized in the lateral direction than those in the experiments with the 2-DOF PAFOs (Table 2). This difference was statistically significant, as shown in Fig 6. Regarding the *COP*_*Avg*_, we found different trends between the 1- and 2-DOF PAFOs. In the 1-DOF PAFO experiment, the larger the duty ratio applied, the more the COP tended to move toward the lateral direction (1-DOF UNP: 51.433, 1-DOF A: 50.688, 1-DOF B: 49.172, 1-DOF C: 48.731). In the case of the 2-DOF PAFO, no significant difference was found between the experiment results, as shown in Table 2 and Fig 6.

### Eversion angle

As eversion is a motion only available in the 2-DOF PAFO, we could only obtain data from experiments using the 2-DOF PAFO. We found that the eversion angle was larger in the experiments with the duty ratio of PAM2 (2-DOF A2, 2-DOF B2, 2-DOF C2, and 2-DOF C3) than in the other experiments (eversion angle sheet of the S1 Dataset).

### Positive power of PAM

The duty ratio and applied *P*_*Avg*_ showed a proportional relationship in PAM1, regardless of the DOF of PAFO. However, in the case of PAM2, the *P*_*Avg*_ was determined by the presence or absence of the duty ratio; however, no correlation was observed. For the 2-DOF PAFO, as PAM2 generates the force together with PAM1, PAM1 has a lower power than for the 1-DOF PAFO, even if the duty ratios are the same (average positive power sheet of the S1 Dataset).

### Stabilogram

**Fig 7:**
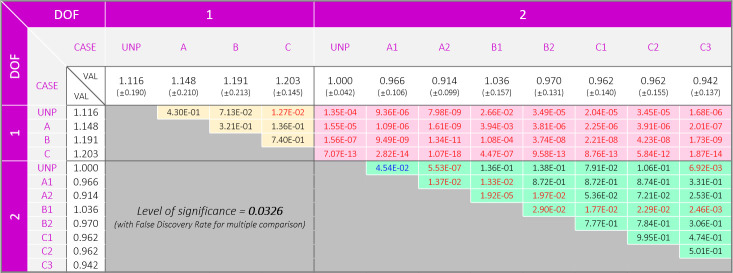
P-table of the stabilogram. The values with blue and red colors represent significant differences of p<0.05 and p<0.0326 for before and after correction, respectively. A significance level of 0.0326 was determined on the basis of FDR. The shadows colored in yellow, pink, and green represent comparisons between 1 DOF − 1 DOF, 1 DOF − 2 DOF, and 2 DOF − 2 DOF, respectively.

The 1-DOF PAFO experiment results showed that the stability in the roll and pitch directions was lower when assistance was generated by applying the duty ratio than that in the unpowered condition (1-DOF UNP). However, in the 2-DOF PAFO, the highest stability was not achieved in the unpowered condition (2-DOF UNP, *δ*_*RMS*_ = 7.339). No significant differences or smaller values were observed in comparison with the values in the unpowered conditions among the 2-DOF PAFO experiments except in 2-DOF B1. We found a significant difference depending on the presence of the subtalar joint, shown with pink shadow in Fig 7.

### Correlation coefficients

Between the experimental results, the Pearson correlation coefficients (*PCCs*) are calculated and shown in Table 3. They are interpreted by following a guide for use in medical research [43]. *COP*_*Avg*_−*δ*_*RMS*_, *P*_*Avg*_−*θ*_*Avg*_ (2-DOF PAFO) and *P*_*Avg*_−*COP*_*Avg*_ (1-DOF PAFO) have a moderate to high relationship with −0.66 ± 0.15, 0.74 ± 0.10 and −0.71 ± 0.16, respectively. However, *θ*_*Avg*_−*COP*_*Avg*_ (2-DOF PAFO) has a negligible to low correlation of 0.25 ± 0.20, and *P*_*Avg*_−*COP*_*Avg*_ (2-DOF PAFO) has no significant relationship between them.

**Table 3:** Average correlation coefficient.

	Pearson (PCC)			Interpretation
	Avg.	*R*^2^	SD	
**COP–Stabilo**	−0.66	0.44	0.15	Moderate to high negative correlation
**Power–Angle (2)**	0.74	0.55	0.10	Moderate to high positive correlation
**Angle–COP (2)**	0.25	0.06	0.20	Negligible to low positive correlation
**Power–COP (1)**	−0.71	0.51	0.16	Moderate to high negative correlation
**Power–COP (2)**	0.18	0.03	0.18	Negligible correlation

Interpretation of correlation was based on the guide for use in medical research [43].

## Discussion

In this study, we applied various assistance techniques for PAFOs with 1- and 2-DOF and compared their stabilities when the direction and magnitude of force applied to the wearer was changed. As a result, we found that 2-DOF PAFOs capable of eversion motion with a subtalar joint ensure high stability.

**Fig 8:**
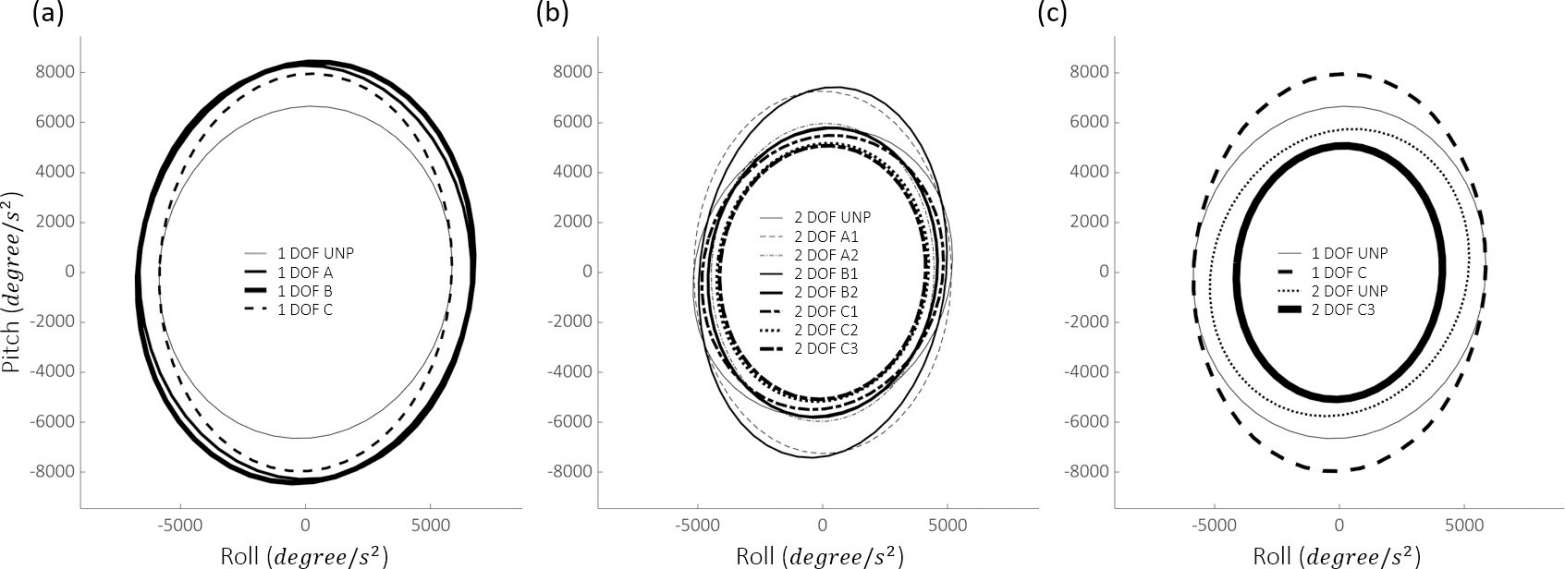
Experimental results of the stabilogram for subject 2. (A) and (B) The results of the experiments that used the assistance techniques demonstrated in Table 1 with 1- and 2-DOF PAFOs, respectively. (C) The stabilogram of the unpowered and maximum output states of 1- and 2-DOF PAFO experiments for comparison. The circles represent 1δ of covariance, which comprised 39% of the data. 2δ (68%) and 3δ (99%) are not shown for clarity of comparison, as they are simple magnifications.

Examination of the stabilogram revealed that most experiments performed with 1-DOF PAFOs demonstrated lower stability (*δ*_*RMS*_) than those with 2-DOF PAFOs, as shown in Table 2 and Fig 7. As shown in Fig 8C, wearing the 1-DOF PAFO in the unpowered condition fundamentally provided lower stability than the 2-DOF PAFO, with a difference of 8.89% (Table 2). This comparison is not accurate without taking into account the applied *P*_*Avg*_; however, the difference in *δ*_*RMS*_ between the experiments with the highest stability among the 2-DOF PAFO experiments (2-DOF A2) and those with the highest stability among the 1-DOF PAFO experiments was 23.78%. As in Fig 7, the comparison of the data between the 1- and 2-DOF PAFO experiments could be achieved on the basis of data with pink shadow, which are significantly different. Notable results were obtained when each DOF was considered separately. In the case of the 1-DOF PAFO experiments, shown in Table 2, the stability worsened when a larger *P*_*Avg*_ was applied to the wearer using a larger duty ratio for PAM1. The data with yellow shadow in Fig 7, which represents comparisons between the 1-DOF PAFO experiments, showed a significant difference in 1-DOF B and C as compared with those in the unpowered condition. We used P-bcs as in the other PAFO experiments, and the *P*_*Avg*_ applied to the wearer resulted in a reduction in metabolic cost and EMG activity of the muscles involved in ankle movement. Therefore, wearing a 1-DOF PAFO may have some advantages; however, the negative correlation implies that it does not improve stability. Therefore, a stable assistance for balanced walking, which prevent wearers from falls because of the absence of a subtalar joint, could be difficult to achieve.

By contrast, in the case of 2-DOF PAFO, a high *P*_*Avg*_ does not cause deterioration of stability and shows no obvious tendencies. Notably, the unpowered condition is also worse than the other cases, as shown in Table 2, and when PAM2 generated force together with PAM1 (2-DOF A1, 2-DOF B1, and 2-DOF C1), the stability was higher in both the roll and pitch directions, regardless of the magnitude of *P*_*Avg*_ when compared with the situations in which only PAM1 was actuated. Therefore, applying a high *P*_*Avg*_ to the wearer, regardless of DOF, does not reduce the stability; however, other confounding factors may exist.

We analyzed the *COP*_*Avg*_ for this purpose. Previous studies showed that indexes based on the COP trajectory play significant roles in the assessment of gait stability, such as the distance between the anteroposterior and mediolateral displacements of the COP, velocity, frequency, or other dimensionless values [44–46]. These values help interpret the balance during gait because the location of the COP under each foot represent the neural control of the muscles related to the ankle joint [47], status of the postural sway during one-leg stance [22], and adjustment of ankle joint moments to change the COM position of the whole body [48]. Among them, the mediolateral movement of COP in the frontal plane can help predict the postural sway in one-leg stance during the stance phase and the risk of injurious falls [44, 49–51]. Thus, we converted the COP trajectory within a sole into the mean value of the mediolateral position during the stance phase, as shown in Table 2.

**Fig 9:**
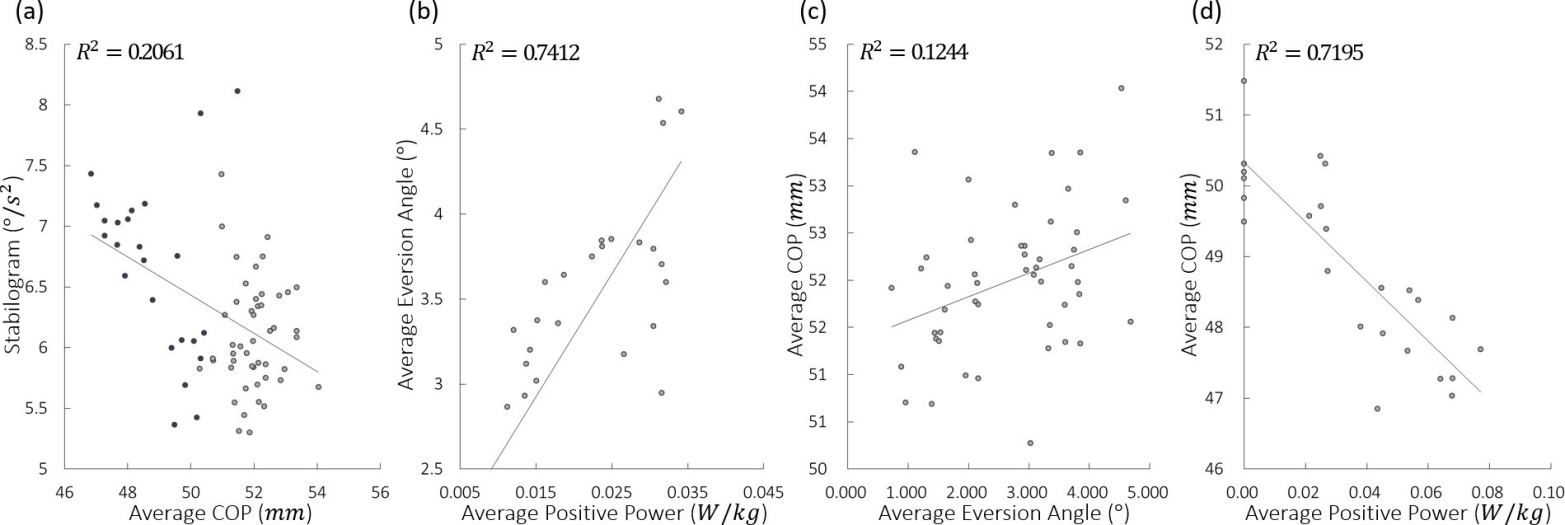
PCC plot of the 4 cases for subject 2 (*COP*_*Avg*_−*δ*_*RMS*_, *P*_*Avg*_−*θ*_*Avg*_, *θ*_*Avg*_−*COP*_*Avg*_, and *P*_*Avg*_−*COP*_*Avg*_). The blue and gray points in (A) represent the mean data of 1- and 2-DOF PAFO experiments, respectively. (B) and (C) The 2- and (D) 1-DOF PAFO experiments.

The result of the experiment conducted with 1-DOF PAFO shows that the greater the *P*_*Avg*_ applied, the more the *COP*_*Avg*_ was located in the lateral position as compared with that in the unpowered condition. In general, when the trajectory of the COP is located in the lateral direction, the extrapolated COP also increases, which reduces the walking stability [25, 52]. Therefore, increasing the duty ratio at 1-DOF PAFO lowers the stability because the *COP*_*Avg*_ is located in a more lateral position. The *COP*_*Avg*_ for the 2-DOF PAFO experiments was small but showed a similar trend as those in the 1-DOF PAFO experiments. When actuating PAM2 with PAM1 as mentioned earlier, the stability was higher. The *COP*_*Avg*_ was located in a more medial direction. Therefore, we conclude that even when wearing PAFOs, keeping the COP in the medial position can help maintain high stability of the individual. The PCC of *COP*_*Avg*_ and *δ*_*Euclid*_ were −0.6607, supporting our conclusion with strong negative correlations, as shown in Table 3. In addition, the blue and gray dots in Fig 9A represent the results of the 1- and 2-DOF PAFO experiments, respectively.

This raises the question of how *P*_*Avg*_ affects stability. We conclude that this effect is caused by the determination of the location of the COP. We calculated the PCC between the *P*_*Avg*_ and *COP*_*Avg*_ by using the results of the 1-DOF PAFO experiments and demonstrated a strong negative correlation of −0.7149. Therefore, the greater the force, the lower the COP will be in the lateral direction. However, the result differed when the 2-DOF PAFO was used.

The difference between the 1- and 2-DOF PAFOs is the presence of a subtalar joint. Therefore, the eversion angle must be evaluated. We added and actuated PAM2 to enhance the eversion motion and found that when a higher duty ratio was applied to PAM2, its size increased (2-DOF A2, 2-DOF B2, 2-DOF C2, and 2-DOF C3), and the eversion motion became stronger, as shown in Table 2. Moreover, the higher the duty ratio applied, the larger is the *P*_*Avg*_ applied by PAM2 to the wearer. We can therefore infer that the greater power PAM2 exerts, the more the eversion can be pulled outward. The PCC between *P*_*Avg*_ and *θ*_*Avg*_ was 0.7441, demonstrating a strong positive correlation (Table 3). Generally, the greater the eversion, the more the COP enters the medial direction. We also calculated the correlation between the two (*θ*_*Avg*_−*COP*_*Avg*_), with a value of 0.2480 showing no significant relationship. However, as shown in Table 2, the COP of 2-DOF A2, 2-DOF B2, 2-DOF C2, and 2-DOF C3, in which PAM2 was actuated, moved more in the lateral direction than those of 2-DOF A1, 2-DOF B1, and 2-DOF C1, in which PAM2 was not actuated. From this, we can predict that the COP were not related to each other. However, in Fig 7, we can observe significantly reduced *δ*_*RMS*_ values between 2-DOF A1–2-DOF A2 (p = 1.37e-5) and 2-DOF B1–2-DOF B2 (p = 2.90e-2), which suggest that PAM2 affects stability. Therefore, as PAM2 was actuated, the eversion motion was stronger, which means that the COP did not move out in the lateral direction and, as a result, did not lead to the deterioration of stability, even though the wearer received assistance.

While PAM2 plays a key role in preserving COP in a stable state through eversion, the primary role of PAM1 is to assist plantar flexion. Unlike in the case of 1-DOF PAFO, the stability did not deteriorate despite the increase in the amount of work that the PAM1 of the 2-DOF PAFO exerted on the wearer. This proves that a high stability can be maintained even if the assistance of the wearer is increased by the addition of DOF and change in the direction of the resultant force to the foot, which is our assumption. However, other existing studies have used 1-DOF PAFO, and in this case, as our study revealed that the greater *P*_*Avg*_ applied to the wearer resulted in lower stability of the wearer. Our experiments were conducted in the same environment, and even in this situation, if the differences in DOF and force direction cause such a large change in stability, they are expected to have greater effects in situations involving rough terrains, slopes, and stairs, which require walking outside the laboratory.

The experiments and results of this study have some limitations. First is that we did not measure EMG activity and metabolic cost. These parameters have primarily been analyzed for experimental validation in previous studies that used 1-DOF PAFOs, whose research focus was the effectiveness of assistance. However, in the field of rehabilitation, those values are not important. As rehabilitation involves training to return to a normal state, subjects who use PAFOs for rehabilitation are patients who wanted to walk normally. These would include patients who have to relearn their walking skills and proprioception, which were lost or deteriorated by accidents, diseases, or aging. As mentioned in the Introduction section, the stabilizing moment is essential in the elderly for training their gait skills so as not to lose their stability [53]. Many exercises can aid in balance training, including the one-leg stance, walking on a narrow path, or weight shifting. In these exercises, PAFOs can help subjects train their evertors and increase the range of motion of eversion by controlling the magnitude and direction of the stabilizing moment in stages [54–56]. After repetition of training with PAFO, subjects can achieve proprioception of their foot during various motions. Hence, the results of this study can be considered meaningful as they show that a 2-DOF PAFO is more suitable for stable assistance.

The second limitation is that we did not measure the temporal parameters during experiments such as step width, stride length, and time. These parameters can affect postural sway, balance, and gait stability, so previous studies used them to evaluate gait quality. For example, the margin of stability (MOS) and extrapolated COM, which are related to balance, require the step width to be calculated [57]. However, an in-shoe pressure sensor was used in this paper, so those parameters cannot be determined. Although the COP trajectory in the foot, which was used in this study, is an important factor for attaining gait stability during one-leg stance, the effect of the parameter is as significant as that of gait stability itself, so an accurate analysis is not possible without it. In the Results section, *θ*_*Avg*_ and *COP*_*Avg*_ have a negligible or low positive relationship (0.25 ± 0.20), which cannot be easily explained because when the eversion motion was enhanced, the COP should be in a more medial position normally. This situation can be interpreted when temporal parameters are measured. We can expect some possible answers for the weak relationship similar to when the eversion motion was enhanced; if the step width is wider than before, then the *COP*_*Avg*_ cannot be in a medial position as much as expected. Thus, these can help us analyze the series of processes in the function of the PAFO in maintaining the wearer’s stability in more detail.

The third limitation is that all the participants were healthy and had no need for rehabilitation. Elderly people and patients with abnormalities in their walking skills or body elements are the potential wearers of 2-DOF PAFOs when these are applied in rehabilitation. Thus, experiments in these people should be conducted following this research to validate the functions of the 2-DOF PAFO because they may respond differently with 2-DOF PAFOs, although the presence of a subtalar joint and its actuation were proven to be useful with healthy people. For the rehabilitation of people in weakened and unstable states, the optimal design and controller of the assistive device for each participant will be needed, which were not realized in this paper. Two axes of the 2-DOF PAFO were calculated on the basis of the average values of the anatomical data from cadavers [10], so they are not perfect for various sizes of participants. Of course, the existence of a subtalar joint was proved, and its actuation can provide the wearer more effective assistance in terms of stability than the existing 1-DOF PAFO, but the outcome can be improved with adjustment of the misalignment achieved. Solving a misalignment between the joints of the wearable robots and the wearers is difficult, but many research studies have attempted to find solutions. A 4-bar linkage for the knee joint is a representative example. We are now considering this mechanism and the Schmidt coupling joint or something else to solve the problem. This might be the focus of our future work. For the controller, we used P-bc for the PAM actuation, but it is not a notable and adjustable method because it does not conduct a tracking control of the predefined or real-time force profile. It induces the maximum contraction of the PAM with given pressure, generating a force that varies with the circumstances at that time. Although a certain duty ratio can generate similar magnitudes of force during experiments, it cannot be adjustable for various sizes and characteristics of wearers and circumstances. The benefit of PAM2 was proved by experiments, but the existence of PAM2 was newly introduced in this paper, so its optimal controller or force profile has not been developed yet, which can be also a direction for our future work. To develop a controller for PAM2, the relationship of the 2 PAMs should be determined because their effects on the moment and rotation of 2 joints are coupled as shown in Fig 2. However, this is not easily solved because the free body diagram of the ankle joints is too complex owing to their structure, stiffness, and force translation model. Thus, some solutions for the free body diagram can be used regardless of difficulty and obtain the exact effect of the portions of the 2 PAMs on each joint or develop a new controller without them. New methods can be created by locating the COP in the medial position as much as possible or using the chaos theory and maximum Lyapunov exponents as objective variables for optimization such as human-in-the-loop [58–60]. In addition, our results can be used for assistive PAFOs to identify the optimal torque profile to reduce the metabolic cost or EMG, which were not measured in this study, and simultaneously secure the gait stability. This indicates that assistance effectiveness can be obtained and PAFOs can also achieve its fundamental purpose.

## Conclusion

We measured the stability of the wearer by applying assistance techniques with various characteristics to 1- and 2-DOF PAFOs and found that the presence of an additional DOF and the direction of force affected stability, regardless of assistance efficiency. Unlike the 1-DOF PAFO, the 2-DOF PAFO has a subtalar joint as the axis of rotation, which enables the eversion motion to preserve *COP*_*Avg*_ in the medial position without being affected by the amount of assistance. The location of the *COP*_*Avg*_ was directly related to the stability of the trunk, the most inferior segment; thus, stability was also preserved. By contrast, the 1-DOF PAFO, which only has the talocrural joint as the axis of rotation, was unable to alter the eversion. As the applied *P*_*Avg*_ increased, the COP moved out in the lateral direction. Consequently, the stability of the trunk deteriorated. This demonstrates the importance of subtalar joints in the use of PAFOs. Further studies are required to prove their importance as indicators of stability and efficiency of assistance.

## Supporting information

S1 Dataset Experimental results of all the participants. This file contains all relative result data during experiments with the participants, which consists of sheets named “Stabilogram,” “Eversion angle,” “Average positive power,” “Average center of pressure,” and “Correlation coefficient.”. Mean and standard deviation of Table 2 was calculated based on the data of this file and graph data of Figs 4 and 6–8 is also included.
(ZIP)

S1 Table Characteristics of assistance techniques. Data for Table 1 is included in this file.
(ZIP)

S2 Table Correlation coefficients between the results. Data for Table 3 and Fig 9 are included in this file.
(ZIP)
